# Recycled Expanded Polystyrene as Lightweight Aggregate for Environmentally Sustainable Cement Conglomerates

**DOI:** 10.3390/ma13040988

**Published:** 2020-02-22

**Authors:** Andrea Petrella, Rosa Di Mundo, Michele Notarnicola

**Affiliations:** Dipartimento di Ingegneria Civile, Ambientale, Edile, del Territorio e di Chimica, Politecnico di Bari, Via E. Orabona, 4, 70125 Bari, Italy; rosa.dimundo@poliba.it (R.D.M.); michele.notarnicola@poliba.it (M.N.)

**Keywords:** recycled expanded polystyrene, cement mortars, safe production, thermal insulation, mechanical resistance

## Abstract

In the present work the rheological, thermo-mechanical, microstructural, and wetting characteristics of cement mortars with recycled expanded polystyrene (EPS) were analyzed. The samples were prepared after partial/total replacement of the conventional sand aggregate with EPS having different grain size and size distribution. Lightness and thermal insulation were relevant features for all the bare EPS composites, despite the mechanical strengths. Specifically, EPS based mortars were characterized by higher thermal insulation with respect to the sand reference due to the lower specific mass of the specimens mainly associated with the low density of the aggregates and also to the spaces at the EPS/cement paste interfaces. Interesting results in terms of low thermal conductivity and high mechanical resistances were obtained in the case of sand-EPS mixtures although characterized by only 50% in volume of the organic aggregate. Moreover, sand-based mortars showed hydrophilicity (low WCA) and high water penetration, whereas the presence of EPS in the cement composites led to a reduction of the absorption of water especially on the bulk of the composites. Specifically, mortars with EPS in the 2–4 mm and 4–6 mm bead size range showed the best results in term of hydrophobicity (high WCA) and no water penetration in the inner surface, due to low surface energy of the organic aggregate together with a good particle distribution. This was indicative of cohesion between the ligand and the polystyrene as observed in the microstructural detections. Such a property is likely to be correlated to the observed good workability of this type of mortar and to its low tendency to segregation compared to the other EPS containing specimens. These lightweight thermo-insulating composites can be considered environmentally sustainable materials because they are prepared with no pre-treated secondary raw materials and can be used for indoor applications.

## 1. Introduction

In recent years, the problems associated with waste management have become very relevant in the frame of a more sustainable model of development and consumption of new resources and energy [1,2,3,4,5,6,7]. The construction industry is one of the activities with the greatest consumption of raw materials together with large production of waste [8,9,10,11,12,13,14]. Specifically, the broad use of plastics in building/construction applications, especially expanded polystyrene (EPS), requires new and low environmental impact approaches for the optimization of the production processes and the reduction of by-products [15,16,17,18]. For this reason, recycling operations can be considered important tasks to increase the sustainability of a material which is converted into a new resource, the so-called secondary raw material. For the purpose, expanded polystyrene is a completely recyclable material, widely used because of cost-effectiveness, versatility and performance characteristics [18,19,20,21]. It is manufactured from styrene monomer using a process during which pentane gas is added to the polymer in order to induce expansion with the following production of spherical beads. EPS is a thermoplastic polymer widely used in many applications (buildings, packaging) due to the relevant features as thermal insulation, durability, lightness, strength, shock absorption, and processability which allow high performance and economic products to be obtained [22,23,24,25,26,27]. EPS is a closed cell material with low water absorption and high resistance to moisture, thus preserving shape, size, and structure after water saturation. EPS resins are widespread polymers in the building/construction field and in civil engineering, usually available in sheet form, shapes or large blocks and used for floor insulation, closed cavity walls, roofs, etc., but also employed in road foundations, pavement construction, impact sound insulation, drainage, modular construction elements, lightweight conglomerates (concretes, mortars), etc. [28,29,30,31,32,33,34]. 

In this work, lightweight cement mortars containing recycled expanded polystyrene (EPS) from the grinding of industrial scraps were prepared with partial or complete replacement of the standard sand aggregate in the mixture, with no addition of additives. A study on the rheological, thermo-mechanical, microstructural, and wetting properties of the samples was conducted. The effect of the aggregate size and size distribution was evaluated and a comparison with specimens based on conventional and/or normalized sand was carried-out. 

The aim was to realize an environmental, sustainable material with low specific mass and thermal insulating properties and characterized by high technical performances in term of hydrophobicity, low water absorption [35,36,37,38,39], and with a low impact manufacturing process. On the contrary to that observed in conventional cement composites, characterized by porosity and hydrophilicity, hydrophobic composites usually show longer durability together with self-cleaning properties [40,41]. Cement structure protection follows standard protocols based on impregnation/coating of external layers by silane or siloxane moieties thus leaving a hydrophilic consolidated concrete composite [41,42]. The addition of polymers to the fresh mixture together with the application of hydrophobic coatings on the hardened artifacts has been shown to lead to a reduction of water penetration thus converting the reference building material to have a hydrophobic or over-hydrophobic nature [43,44]. In the present research, the conglomerate did not show any coating on the surface and the whole mass was modified, for this reason the side and the fracture surfaces were investigated. 

These lightweight thermo-insulating composites can be considered environmentally sustainable materials for indoor non-structural artifacts because they are prepared with non-pre-treated secondary raw materials and with a cheap route since complex techniques of production are not required. These treatments and processes would, however, be more effective in the case of production on a larger scale. 

## 2. Materials and Methods

### 2.1. Preparation of the Mortars

Cement mortars were prepared with CEM II A-LL 42.5 R (Buzzi Unicem (Casale Monferrato, Italy)) [45]. Normalized sand (~1700 g/dm^3^, 0.08–2 mm) was purchased by Societè Nouvelle du Littoral (Leucate, France), whereas sieved sand was used as aggregate in three specific size fractions (1–2 mm, 2–4 mm and 4–6 mm) [46,47]. Recycled expanded polystyrene (EPS), resulting from grinding of industrial scraps, was employed in three specific size fractions (1–2 mm, 2–4 mm, and 4– 6 mm). The specimens were prepared with a 0.5 W/C ratio and 40 mm × 40 mm ×160 mm prisms were obtained for the flexural/compressive tests, while cylinders (diameter = 100 mm; height = 50 mm) were prepared for the thermal tests. In the case of the mechanical tests the samples were cured in water for 7, 28, 45, and 60 days, while in the case of the thermal tests the samples were cured in water for 28 days. 

The reference was prepared with normalized sand [46] and named Normal. EPS was added into the conglomerate with a partial or complete replacement of the standard sand aggregate which was made on volume basis rather than on weight basis [48,49,50] due to the low specific mass of polystyrene. The samples (excluding the Normal) were prepared with a volume of aggregate of 500 cm^3^. Table 1 and Table 2 show the composition of the aggregate and of the relative mortars. 

Total sand replacement was carried out with EPS grains in the 1–2 mm (30 g/dm^3^), 2–4 mm (15 g/dm^3^) and 4–6 mm (10 g/dm^3^) bead size range and EPS2, EPS3, and EPS4 samples were obtained as reported in Table 1 and Table 2. Another reference, named Sand, prepared with sand size in the range of 1–2 mm (50%), 2–4 mm (25%), and 4–6 mm (25%), was compared to the EPS specimens. A Sand-EPS sample was prepared after replacement of the 50% sand volume with 4–6 mm EPS grains (Sand/EPS). 

### 2.2. Rheological, Thermal and Mechanical Characterization

The flow-test allowed the evaluation of the rheological properties of the fresh conglomerates [51]. An ISOMET 2104, Applied Precision Ltd (Bratislava, Slovakia), was used to determine the thermal conductivity (λ) and the thermal diffusivity (α) of the specimens by production of a constant thermal flow by a heating probe applied on the sample surface. The temperature was recorded over time and λ and α were obtained after evaluation of the experimental temperature compared with the solution of the heat conduction equation [52]. The flexural and compression tests were carried out by the use of a MATEST device (Milan, Italy). Flexural tests were carried out on six prisms (40 mm × 40 mm ×160 mm) by applying a load with a 50 ± 10 N/s rate, while compression strengths were obtained on the resulting semi-prisms by applying a load with a 2400 ± 200 N/s rate [46]. 

### 2.3. Measurements of the Contact Angle and of the Water Absorption

In the present research, an investigation on the side surface and on the inner surface of the cement conglomerates was carried out by contact angle measurements. After deposition of at least fifteen drops (5 µL) of water onto the surface of each specimen, it was shown that the behavior of three representative points (point 1, 2, and 3) summarized the behavior of all the drops. A Premier series dyno-lite (Taiwan) portable microscope and background cold lighting was used to study the time evolution of the drop, with a rate ranging of 30 frames per second. In the case of non- static drop, determined by water absorption, the image sequences were analyzed by Image J software (version 1.8.0, National Institute of Health, Bethesda, MD, USA) in order to measure the variation of the contact angle and of the drop height.

### 2.4. SEM/EDX and Porosimetric Analyses

An electron microscope FESEM-EDX Carl Zeiss Sigma 300 VP (Carl Zeiss Microscopy GmbH (Jena, Germany)) was used to characterize the morphology and the chemical composition of the samples which were applied onto aluminum stubs and sputtered with gold (Sputter Quorum Q150 Quorum Technologies Ltd (East Sussex, UK)) before the test. In this respect, the composition of the normalized sand was: C (4%), O (52%), Si (35%), Ca (2%), the composition of the sieved sand was: C (10%), O (45%), Ca (45%), polystyrene composition was: C (30%), O (70%), the composition of the cement paste was: C (4.2%), O (40%), Si (7.6%), Ca (44%), Fe (1.5%), Al (2.5%). Ultrapyc 1200e Automatic Gas Pycnometer, Quantachrome Instruments (Boynton Beach, FL, USA) was used for the porosimetric measurements and helium was used to penetrate the material pores.

## 3. Results and Discussion

**Figure 1:**
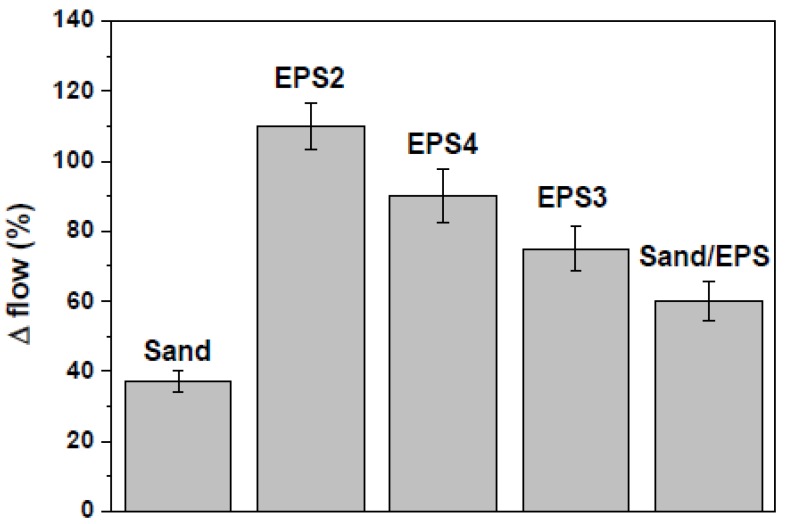
Flow-test results.

Flow data of the non-consolidated samples are reported in Figure 1 and were obtained after measuring the diameters of the mixture before and after the test [51]. The flow of a sample is represented by the percentage increase of the diameter over the base diameter.

**Figure 2:**
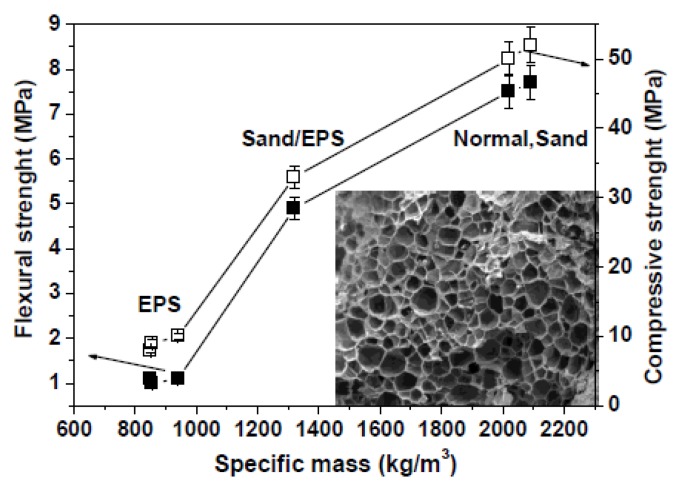
Flexural and compressive strengths of the samples (28 days curing). The label EPS (expanded polystyrene) represents EPS 2, EPS3, and EPS4. White squares represent the compressive strengths, while black squares represent flexural strengths. In the inset: inner porosity of an EPS bead (SEM image).

The Sand sample showed a higher flow (+35%) with respect to the Normal sample due to the absence of finer aggregates. The EPS specimens were more fluid than both references and specifically with respect to the normalized mortar (Normal). This behavior may be ascribed to the low surface energy, the low roughness (smooth surface), the hydrophobic features (synthetic organic polymer) and the low density of the EPS particles (10–30 g/dm3 with respect to 1700 g/dm^3^ of sand) which may induce aggregate segregation in a cement conglomerate. The lower fluidity of EPS3 (+126%) with respect to EPS2 (+174%) and EPS4 (+150%) is likely due to a better compaction of the aggregates in the mixture (better distribution of the granules), while in the case of the Sand/EPS specimen, the presence of the inorganic aggregate contributed to a reduction of fluidity (Figure 1). In Figure 2 and Table 3 the flexural and compressive strengths of the samples are reported as a function of the specific mass. The Sand sample showed slightly higher mechanical strengths than the Normal sample due to the presence of larger size aggregates which contribute to the increase of the specific mass. The addition of EPS determined the formation of voids in the composite with a sensible reduction of the specific mass of the mortars (Table 2) which depends not only on the matrix and polymer characteristics (foaming structure of EPS), but also on the interface properties [53,54,55]. For this reason, after total replacement of the sand volume, a decrease was observed of the mechanical strengths of the conglomerates, this effect is ascribable to the low density/high porosity of the EPS beads (inset Figure 2) and to the voids created by the aggregate at the cement/EPS interface during mixing [53,54]. In fact, the porosity of these samples is approximately two times higher than the references (Table 2). For this purpose, the flexural and the compressive resistances of EPS2, EPS3, and EPS4 samples were approximately ~80% lower than the references, with compressive strengths passing from nearly 50 MPa to less than 10 MPa as the specific mass was lowered from 2100 to 900 kg/m^3^. After substitution of 50% of the sand volume with EPS beads (Sand-EPS), an increase was observed of the mechanical strengths with respect to the EPS specimens. In fact, the flexural strength decrease was approximately 25% with respect to both references, while the compressive strength was 25%–30% lower than the references.

**Figure 3:**
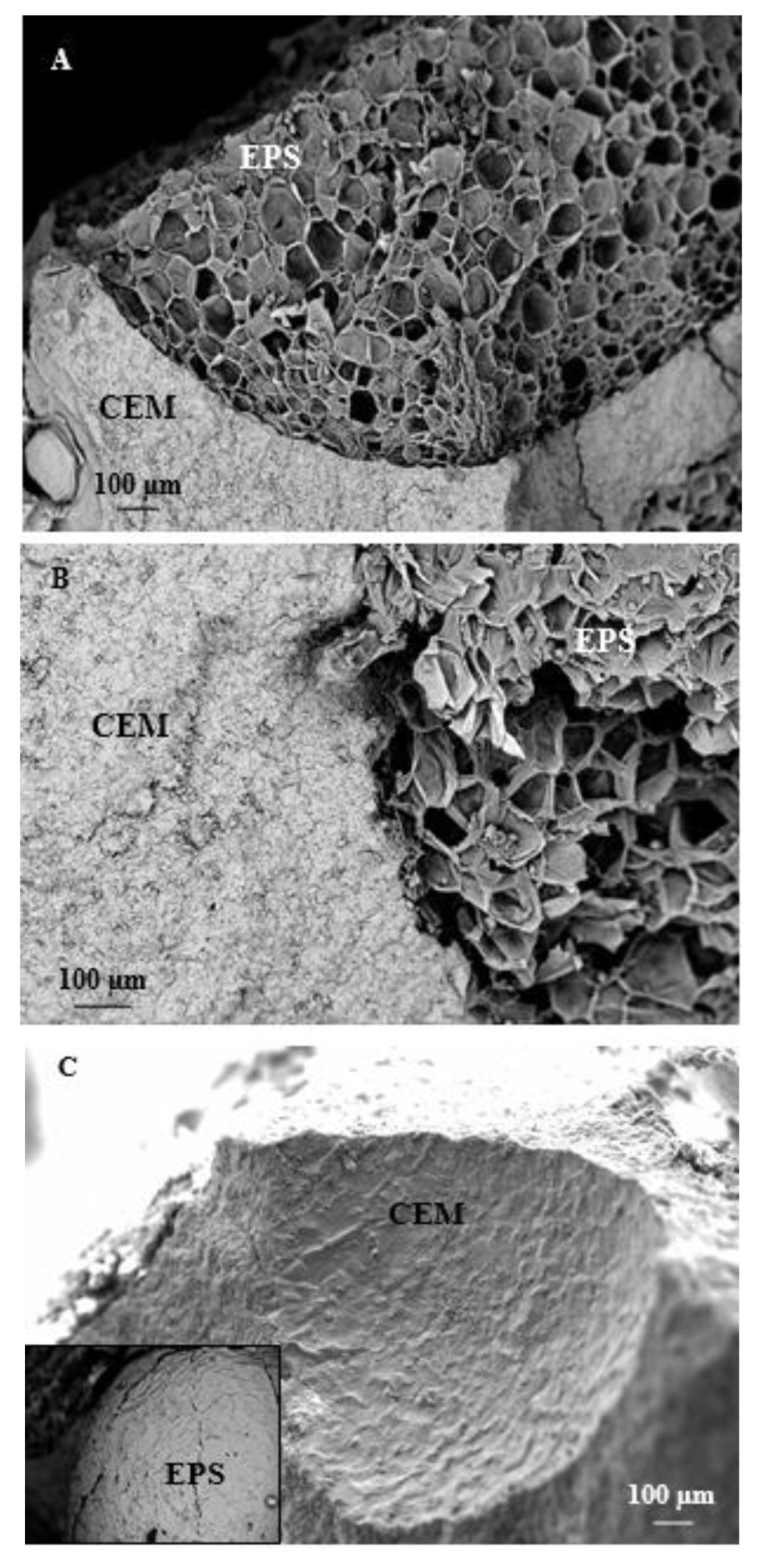
(**A**) SEM image of the cement paste/EPS interface in the EPS3 sample. (**B**) SEM image of the cement paste/EPS interface in the EPS4 sample. (**C**) SEM image of the cement paste/EPS interface in the EPS2 sample, in the inset an image of the de-bonded EPS bead.

The EPS mortars did not show a flexural brittle behavior which can be observed in the sand specimens (Normal and Sand), but the rupture was more gradual and the mortars containing 100% EPS volume did not show a separation of the two parts [56,57]. The Sand-EPS sample, characterized by 50% of sand and 50% of EPS, showed a semi-brittle behavior. As in the former case, the compressive failure of the EPS2, EPS3, and EPS4 mortars was gradual with high energy absorption because of the load retention after rupture without collapse [56,58,59]. As expected, the reference samples showed a typical brittle failure. It was observed that most of the aggregates of the EPS3 and EPS4 specimens sheared off along the failure plane (Figure 3A,B), on the contrary no damage was observed to most of the aggregates in EPS2 mortar and some of the EPS2 beads were de-bonded from the matrix (Figure 3C).

From these results, it can be concluded that the bond between the EPS2 aggregate and the cement paste was weaker than the failure strength of the aggregate (poor EPS adhesion to the cement paste), while the bond between the EPS aggregate and the cement paste in EPS3 and EPS4 samples was stronger (better EPS adhesion to the cement paste) than the failure strength of the polystyrene granules [33,60].This effect was noticed in particular on the EPS3 sample (Figure 3A). The latter result is indicative of better cohesion between the aggregate and the cement paste. Thus, EPS3 exhibited higher compaction which packs the aggregate particles together, so as to increase the specific mass of the mortar, and this also explains the lower percentage flow with respect to the other samples which resulted in more fluid and with a higher tendency to segregation [20] (see Figure 1). 

**Figure 4:**
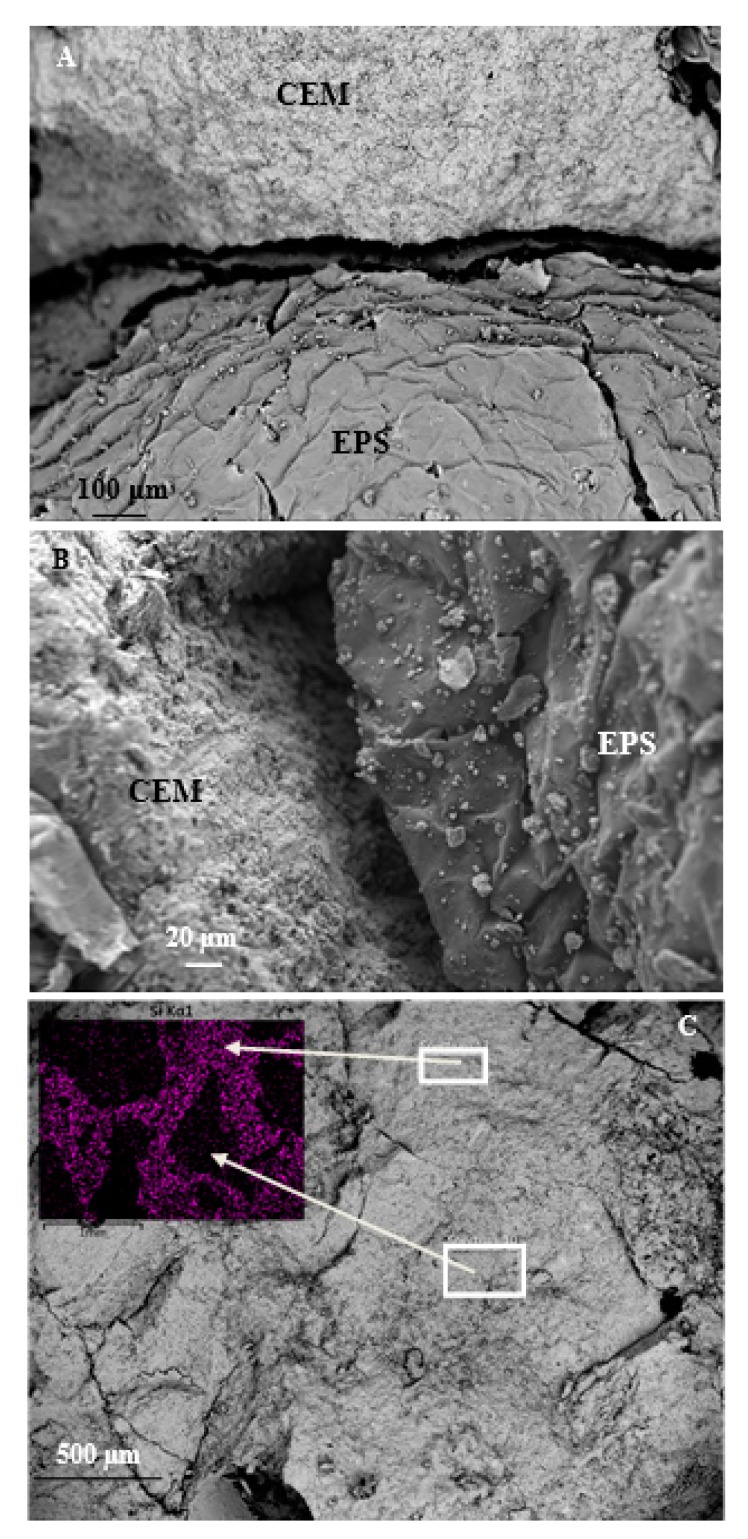
(**A**,**B**) SEM images of the cement paste/EPS interface in the EPS2 sample. (**C**) SEM image of the normalized mortar and, in the inset, EDX map relative to the Si distribution in the sample.

The lower specific mass of the EPS2 sample can be demonstrated by large voids at the ligand/aggregate interface, with length comparable to EPS beads and 20–30 micron width, this effect was ascribed to the mentioned poor adhesion of the beads to the cement paste (Figure 4A,B). This result was also observed in the EPS3 sample, but in the latter case the adhesion of the sheared-off particles to the cement paste was better thus demonstrating the higher specific mass of this type of lightweight mortar. Moreover, from Figure 4C, the perfect adhesion of the sand to the cement paste is evident. In fact, from the map relative to the Si element, which is barely present in the limestone, a negligible separation between the sand and the ligand can be observed ascribed to a favorable adhesion.

**Figure 5:**
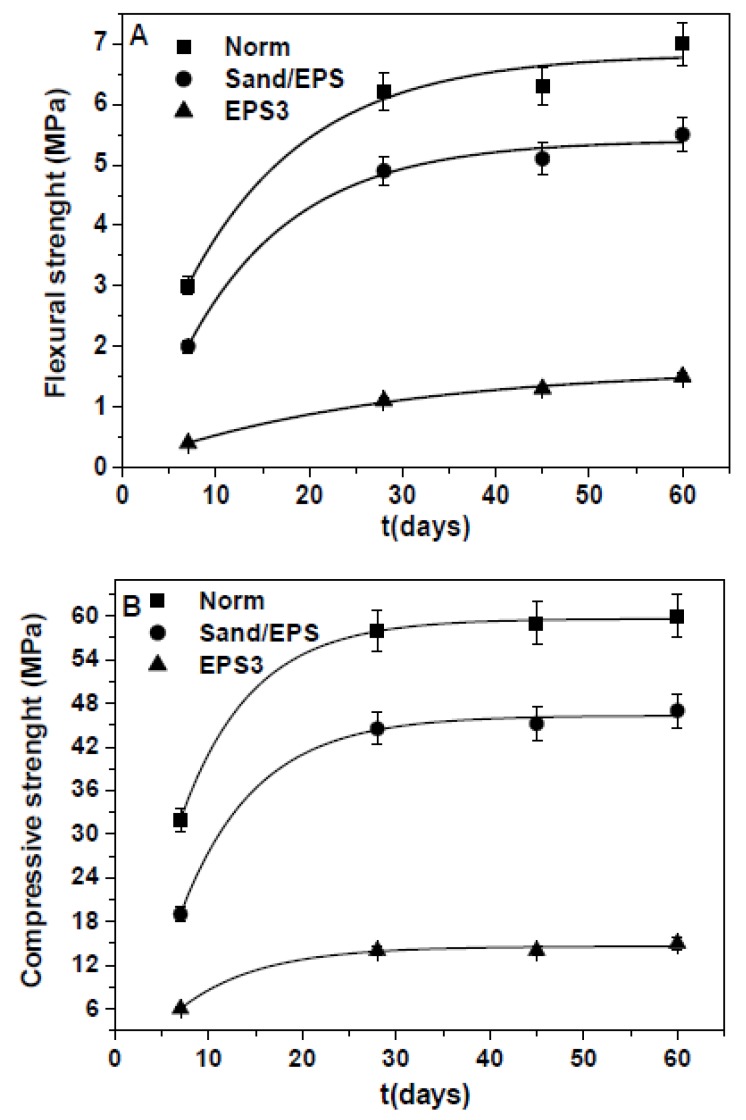
Flexural (**A**) and compressive (**B**) strengths of the samples over time.

The time variation of the flexural and compressive strengths of the Normal sample, of the EPS3 and Sand/EPS samples is reported in Figure 5 where an increase of the resistances can be observed with stabilization after 45 days. At 60 days, the values did not sensibly change thus demonstrating stability of the materials on consideration of the specific water curing/conservation conditions of the conglomerates. 

**Figure 6:**
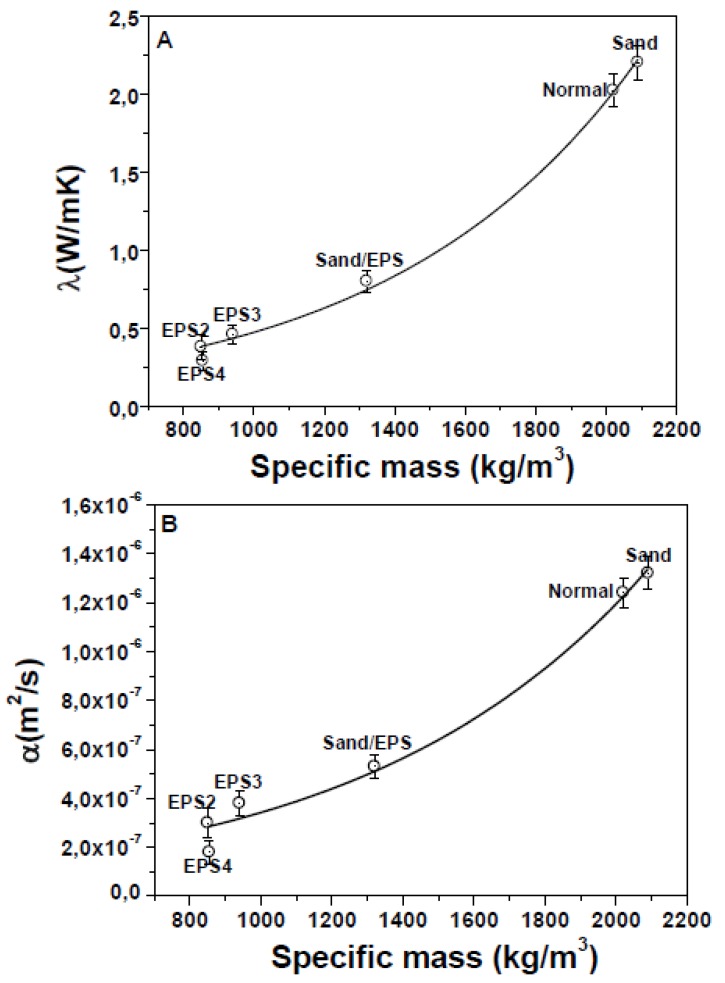
(**A**) Thermal conductivity and (**B**) thermal diffusivity of the samples.

EPS based mortars showed lower thermal conductivities and diffusivities than the sand references (Figure 6). This result can be ascribed to the lower specific mass of the specimens due to the low density of the organic aggregates [61,62] (see inset Figure 2) together with the mentioned voids at the EPS/ligand interface which limit heat transport in the composite. Specifically, the thermal conductivities of the bare EPS specimens were ~80% lower than the references. The best results were obtained in the case of the EPS4 specimen (0.29 W/mK) due to the lowest specific mass. Intermediate values (0.8 W/mK) were obtained in samples with 50% of EPS (Sand/EPS sample). Thermal conductivity and diffusivity data showed an exponential decrease with the decrease of the conglomerates specific mass. 

**Figure 7:**
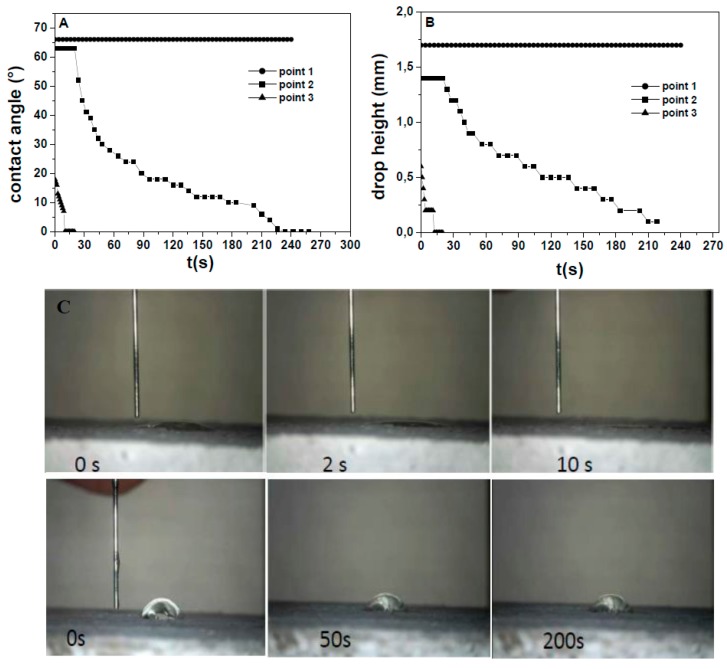
(**A**) Contact angle and (**B**) height variation over time for water drops deposited on representative points of the side surface of the normalized mortar (Sand). (**C**) Optical microscope images (down: point 1 drop, top: point 3 drop).

**Figure 8:**
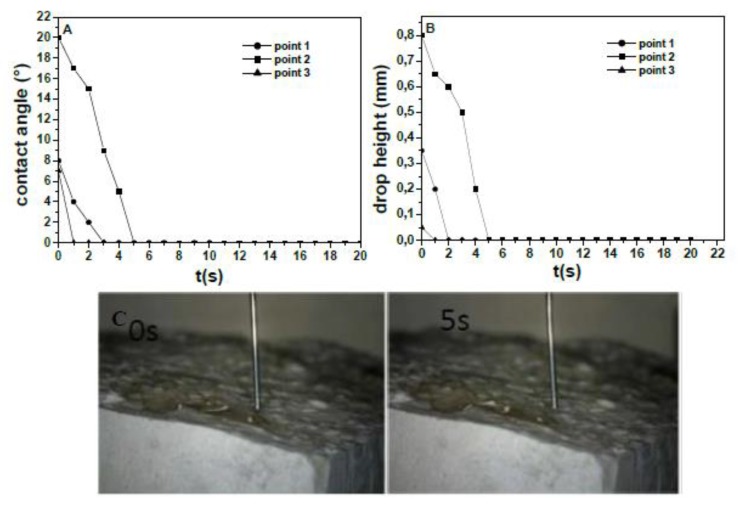
(**A**) Contact angle and (**B**) drop height for representative points of the fracture surface of the normalized mortar (Sand). (**C**) In the optical microscope image: point 2 drop.

The wetting characterization of the side surface (Figure 7) and of the inner surface (Figure 8) of the Normal sample was carried out. Figure 7A,B shows the time evolution of the water contact angle (WCA) and of the drop height for the side surface of the Sand sample. A hydrophilic character (WCA < 90°) [35] was observed although different behavior on various points of observation was detected. Fast WCA decrease and full penetration occurred in few seconds at point 3, slower but full water absorption occurred at point 2, whereas higher WCA and negligible water absorption were observed in the case of point 1. Figure 7C shows the pictures relative to the drop behavior. The side surface of the reference mortar based on normalized sand (Normal) showed similar features. It is worth highlighting that the possibility of detecting and quantifying the spatially non-homogeneous behavior of a surface/material like these is a specific benefit of the spatially resolved evaluation of wettability and absorption made by this technique (the drop volume is 5 µL), which cannot be achieved with water permeability or capillary absorption measurements.

Figure 8A,B shows the wetting parameters relative to the fracture surface. The inner surface resulting from the mechanical breakage can be considered more representative of the composite features because it is a section of the sample showing every component of the mixture. It shows an open porosity characterized by high roughness and a visible distribution of the aggregates, as opposed to what is observed on the side surface. Specifically, the results obtained on every point of the observation were similar. A fast decrease of the water contact angle and of the drop height was observed at every point (Figure 8C). On the contrary to what was observed on the side surface, WCA was lower, thus the fracture surface can be generally considered super-hydrophilic (WCA ~0–5 [35,63] and fast absorbent. As in the former case, similar results were observed on the inner surface of the Normal sample.

**Figure 9:**
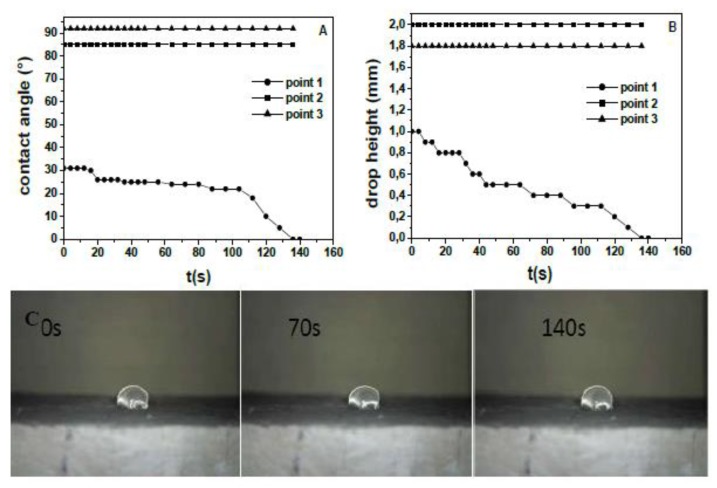
(**A**) Contact angle and (**B**) drop height for representative points of the side surface of the EPS3 mortar. (**C**) In the optical microscope image: point 2 drop.

**Figure 10:**
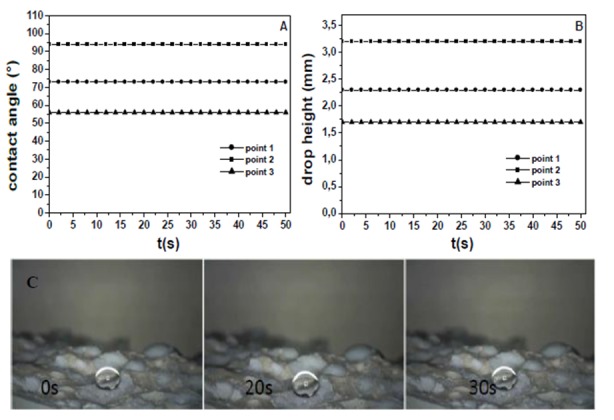
(**A**) Contact angle and **(B**) drop height for representative points of the fracture surface of the EPS3 mortar. (**C**) In the optical microscope image: point 2 drop.

The wetting characterization of the EPS3 mortar, with EPS grains in the 2–4 mm (50%) and 4–6 mm (50%) bead size range, is reported in Figure 9 and Figure 10. As described above, EPS totally replaced the sand volume. Figure 9A,B shows the time evolution of the water contact angle (WCA) and of the drop height on the side surface of the sample. Different trends were observed. A slow but full water absorption occurred at point 1, higher WCA and negligible water absorption were observed in the case of points 2 and 3, the latter with WCA ≥ 90°. In the present case, the side surface resulted in being more hydrophobic than the references. 

Figure 10A,B shows the time evolution of the water contact angle (WCA) and of the drop height on the fracture surface of the EPS3 sample. In this case, the drop was stable for all the observation time. Figure 10 also shows a picture of the drop after deposition onto the specimen surface (point 2) which resulted in being hydrophobic with high WCA (WCA > 90°) [35]. The latter result was confirmed after a drop deposition onto an EPS slab or onto bare EPS beads, specifically in the first case WCA was approximately 99°, while higher in the second (100–102°) probably due to the beads’ curvature. The WCA was higher on bare beads with respect to the EPS in the mixture because of the absence of contamination from the hydrophilic cement paste [64,65]. For this purpose, after deposition onto cement paste regions of the EPS3 sample (points 1 and 3), hydrophilic behavior but negligible water absorption were observed. This latter result is ascribed to the hydrophobic and non- absorbing effect of EPS whose sites decrease the mean surface energy of the sample making the presence of the porous and hydrophilic cement regions ineffective [64,65]. 

**Figure 11:**
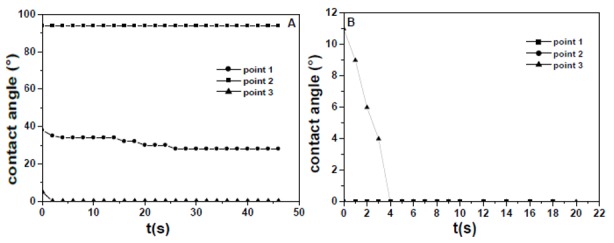
Contact angle for representative points of the fracture surface of (**A**) EPS4 and (**B**) EPS2 mortars.

The wetting characterization of the fracture surface of the EPS4 mortar, with EPS grains in the 1–2 mm (25%), 2–4 mm (25%), and 4–6 mm (50%) bead size range, is reported in Figure 11A, while the results obtained on the side surface were similar to those of the EPS3 specimen. The fracture surface is hydrophobic in the domain of the polystyrene beads (point 2) and hydrophilic in the domain of the cement paste (point 3) because the drop was deposited onto a hydrophilic and absorbent surface. As a matter of fact, the latter result represents a difference between the fracture surface of this sample and the fracture surface of the former composite (EPS3). 

Wetting characterization of the fracture surface of the EPS2 mortar, with EPS grains in the 4–6 mm (100%) bead size range, is reported in Figure 11B and in this case, the results obtained on the side surface of this sample were similar to those observed in the case of the former EPS specimens. In the case of the fracture surface, a hydrophilic character was observed at every point of observation, with very low water contact angle and fast water absorption. 

Thus, EPS3 is the specimen with the lowest water drop absorption. This may be due to a more efficient organization of the aggregate particles with open spaces (spheroidal microcavities) between larger particles filled with smaller size EPS beads [49,66], which leads to better behavior of the composite. This specimen indeed shows the highest specific mass and lowest porosity among the EPS specimens, reasonably as a consequence of a better aggregate compaction (evidenced by the lowest flow). This property on the one hand results in a slight reduction of the thermal insulation performances, but on the other hand makes the composite definitely less subject to water ingress. The importance of optimizing the level of compaction, by adjusting the size distribution of the EPS aggregates, is due to the relatively large size of the initial EPS beads, which results in the formation of too large channels of the cement matrix among the aggregates in the hardened artifacts.

Hence, once properly distributed in size, EPS beads can represent suitable aggregates in cement- based artifacts both for lightening/insulating and water proofing purposes. Such a double advantage arises from the peculiar combination of low density and low surface energy of this plastic matter as already shown by using other polymeric aggregates, such as granulated rubber from end-of-life tires [53].

## 4. Conclusions

In the present work an investigation into the rheological, thermo-mechanical, microstructural and wetting characteristics of cement mortars containing recycled expanded polystyrene (EPS) was carried out. The samples were prepared after partial/total replacement of the conventional sand aggregate with EPS having different grain size and size distribution. The experimental results may be summarized as follows:

- EPS samples resulted in having more fluid than the references, in particular the sample characterized by EPS grains in the 2–4 mm (50%) and 4–6 mm (50%) bead size range (EPS3) had the most plastic with good particle distribution and cohesion between the ligand and the organic aggregates as also observed in microstructural and porosimetric detections.
- The mechanical resistances of the EPS samples were lower with respect to the controls due to the lower specific mass. An increase of the strengths was observed with a stabilization after 45 days. At 60 days the values did not sensibly change thus demonstrating stability of the materials in consideration of the specific water curing/conservation conditions of the conglomerates.
- The EPS based mortars showed lower thermal conductivities and diffusivities as compared to the references based on sand due to a lower density ascribed to the low density of the aggregates and to the spaces at the EPS/cement paste interface.
- Interesting results in term of high mechanical resistances and low thermal conductivity were obtained in the case of sand-EPS mixtures.
- Reference sand-based mortars showed hydrophilicity (low WCA) and high water penetration, particularly on the fracture surface of the composites, conversely to what was observed in the case of EPS samples which were generally more hydrophobic and less water absorbent. The best results (high WCA and negligible water penetration on the fracture surface) were obtained with the EPS3 sample. This property was ascribed to the low surface energy of the organic aggregate combined with its better particle distribution and compaction within the hydrophilic domains of the cement paste in the composite.
- These lightweight thermo-insulating composites may be used in the building industry as non-structural components, with specific reference to indoor applications (panels, plasters). Moreover, the conglomerates can be considered environmentally sustainable because they are prepared with secondary raw materials (recycled EPS) and are cost-effective because a cheap preparation route was used since the renewable aggregates were not pre-treated and a complex technique of production was not required.

## Figures and Tables

**Table 1:** Composition of the aggregates in the composites.

Normal	Normalized Sand		
Sand	sand (1–2 mm) 25%	sand (2–4 mm) 25%	sand (4–6 mm) 50%
Sand-EPS	sand(1–2 mm) 25%	sand(2–4 mm) 25%	EPS (4–6 mm) 50%
EPS 2			EPS (4–6 mm) 100%
EPS 3		EPS (2–4 mm) 50%	EPS (4–6 mm) 50%
EPS 4	EPS (1–2 mm) 25%	EPS (2–4 mm) 25%	EPS (4–6 mm) 50%

**Table 2:** Composition of the mortars.

Sample	Cement (g)	Water (cm^3^)	Sand Volume (cm^3^)	EPS Volume (cm^3^)	ρ (Kg/m^3^)	Porosity %
Normal	450	225	810	0	2020	22
Sand	450	225	500	0	2090	20
Sand-EPS	450	225	250	250	1320	32
EPS 2	450	225	0	500	850	49
EPS 3	450	225	0	500	940	42
EPS 4	450	225	0	500	855	48

**Table 3:** Mechanical strengths (28 days curing) of the samples.

Sample	ρ (Kg/m^3^)	R_F_ (MPa)	R_C_ (MPa)
Normal	2020	7.5	50
Sand	2090	7.7	52
Sand-EPS	1320	4.9	33
EPS 2	850	1.1	8
EPS 3	940	1.1	10
EPS 4	855	1	9
